# Preliminary results of the initial human clinical trial of focused ultrasound to reposition kidney stones

**DOI:** 10.1186/2050-5736-3-S1-O71

**Published:** 2015-06-30

**Authors:** Michael Bailey, Barbrina Dunmire, Bryan Cunitz, Jonathan Harper, Franklin Lee, James Lingeman, Mathew Sorensen

**Affiliations:** 1 University of Washington, Seattle, Washington, United States; 2 Indiana University, Indianapolis, Indiana, United States

## Background/introduction

Ultrasonic propulsion is a new technology we have developed which uses focused ultrasound to transcutaneously reposition kidney stones. Two applications are expelling small stones or fragments and dislodging obstructing stones. We report preliminary, investigative findings from the first use of this technology in humans.

## Methods

There are three arms of the study: de novo stones, post-lithotripsy fragments, and large stones within the preoperative setting. A pain questionnaire is completed immediately prior to and following propulsion. A maximum of 40 push attempts are administered on either low (50 V) or high (90 V) power settings. Movement is classified as no motion, movement with rollback (2-3 mm), or movement to a new location (> 3 mm) (Table 1).

**Table 1:** **Ultrasonic Propulsion of Kidney Stones - Preliminary Results.** All subjects treated with a combination of 50 V and 90V output.

Study Arm	Patient #	Number of Stones	Stone size (mm)	Motion Grading			Total Push Bursts
				2-3mm	>3mm	Passed stones	
De-Novo Stones	1	3	2-3	**5 (19%)**	**0**	**N**	27
	2	5	2-3	**16 (41%)**	**0**	**N**	39
	3	2	3	**1 (4%)**	**0**	**N**	23
Post-Lithotripsy	4	2	2	**23 (62%)**	**5 (13%)**	**Y**	37
	5	5	2-3	**21 (53%)**	**2 (5%)**	**Y**	40
	6	Multiple	< 2	**12 (30%)**	**4 (10%)**	**Y**	40
	7	Multiple	2-5	**5 (13%)**	**20 (50%)**	**unclear**	40
Pre-operative	8	2	7	**7 (26%)**	**0**	**N/A**	27

## Results and conclusions

Eight subjects have been enrolled and undergone ultrasonic propulsion to date. None of the subjects reported pain associated with the treatment. Subjects did report a mild warming of the skin with high (90 V) power pushes; no sensation was reported with low (50 V) power pushes. A summary of push movement results is provided in Table 1. Stones were visualized and repositioned in all subjects. One subject in the post-lithotripsy arm passed two small stones immediately following treatment. At least three post-lithotripsy subjects reported passage of multiple small fragments within two weeks of treatment. In five subjects, ultrasonic propulsion identified a collection of stones previously characterized as a single stone on KUB and ultrasound. Pre-operatively, one of two 7 mm stones was move before surgery. In surgery, it appeared one stone was firmly attached to Randall’s plaque and the other stone had been detached from Randall’s plaque and a 4 mm piece moved into the ureter. There have been no treatment related adverse events reported with mean follow-up of 3 months.

This is the first report of the successful repositioning of kidney stones in humans using ultrasound. Treatment was therapeutic in four subjects and provided diagnostic information in five. Subjects who did not have significant movement were the first patients and in the de novo arm. To date, there have been no reports of pain or adverse events associated with this treatment.

